# Standardised Outcomes in Nephrology—Polycystic Kidney Disease (SONG-PKD): study protocol for establishing a core outcome set in polycystic kidney disease

**DOI:** 10.1186/s13063-017-2298-4

**Published:** 2017-11-23

**Authors:** Yeoungjee Cho, Benedicte Sautenet, Gopala Rangan, Jonathan C. Craig, Albert C. M. Ong, Arlene Chapman, Curie Ahn, Dongping Chen, Helen Coolican, Juliana Tze-Wah Kao, Ron Gansevoort, Ronald Perrone, Tess Harris, Vicente Torres, York Pei, Peter G. Kerr, Jessica Ryan, Talia Gutman, Martin Howell, Angela Ju, Karine E. Manera, Armando Teixeira-Pinto, Lorraine A. Hamiwka, Allison Tong

**Affiliations:** 1 Department of Nephrology, Princess Alexandra Hospital, 199 Ipswich Road, Woolloongabba, Brisbane, QLD 4102 Australia; 2 Australasian Kidney Trials Network, University of Queensland, Brisbane, Australia; 3 Translational Research Institute, Brisbane, Australia; 4 Department of Nephrology and Clinical Immunology, Tours Hospital, University Francois Rabelais, INSERMU1246, Tours, France; 5 Centre for Transplant and Renal Research, Westmead Institute for Medical Research, The University of Sydney, Sydney, Australia; 6 Sydney School of Public Health, The University of Sydney, Sydney, Australia; 7 Centre for Kidney Research, The Children’s Hospital at Westmead, Sydney, Australia; 8 Academic Nephrology Unit, Department of Infection Immunity & Cardiovascular Disease, University of Sheffield, Sheffield, UK; 9 Department of Medicine, The University of Chicago, Chicago, USA; 10 Division of Nephrology, Seoul National University Hospital, Seoul, South Korea; 11 Kidney Institute, Department of Nephrology, Shanghai Changzheng Hospital, Second Military Medical University, Shanghai, China; 12 Polycystic Kidney Disease Foundation of Australia, Sydney, Australia; 13 School of Medicine, Fu Jen Catholic University, Taipei, Taiwan; 14 Department of Internal Medicine, National Taiwan University Hospital, Taipei, Taiwan; 15 Faculty of Medical Sciences, University Medical Center Gronigen, Gronigen, The Netherlands; 16 Division of Nephrology, Tufts University School of Medicine, Boston, USA; 17 Polycystic Kidney Disease International, London, UK; 18 Department of Nephrology and Hypertension, Mayo Clinic, Rochester, USA; 19 Division of Nephrology and Division of Genomic Medicine, University of Toronto, Toronto, Canada; 20 Department of Nephrology, Monash Medical Centre and Monash University, Melbourne, Australia; 21 Division of Nephrology, Albert Children’s Hospital, University of Calgary, Calgary, Canada

**Keywords:** Core outcome set, Outcomes research, Patient-centred outcomes clinical trials, Chronic kidney disease, Autosomal dominant polycystic kidney disease

## Abstract

**Background:**

Autosomal dominant polycystic kidney disease (ADPKD) is the most common potentially life threatening inherited kidney disease and is responsible for 5–10% of cases of end-stage kidney disease (ESKD). Cystic kidneys may enlarge up to 20 times the weight of a normal kidney due to the growth of renal cysts, and patients with ADPKD have an increased risk of morbidity, premature mortality, and other life-time complications including renal and hepatic cyst and urinary tract infection, intracranial aneurysm, diverticulosis, and kidney pain which impair quality of life. Despite some therapeutic advances and the growing number of clinical trials in ADPKD, the outcomes that are relevant to patients and clinicians, such as symptoms and quality of life, are infrequently and inconsistently reported. This potentially limits the contribution of trials to inform evidence-based decision-making. The Standardised Outcomes in Nephrology—Polycystic Kidney Disease (SONG-PKD) project aims to establish a consensus-based set of core outcomes for trials in PKD (with an initial focus on ADPKD but inclusive of all stages) that patients and health professionals identify as critically important.

**Methods:**

The five phases of SONG-PKD are: a systematic review to identify outcomes that have been reported in existing PKD trials; focus groups with nominal group technique with patients and caregivers to identify, rank, and describe reasons for their choices; qualitative stakeholder interviews with health professionals to elicit individual values and perspectives on outcomes for trials involving patients with PKD; an international three-round Delphi survey with all stakeholder groups (including patients, caregivers, healthcare providers, policy makers, researchers, and industry) to gain consensus on critically important core outcome domains; and a consensus workshop to review and establish a set of core outcome domains and measures for trials in PKD.

**Discussion:**

The SONG-PKD core outcome set is aimed at improving the consistency and completeness of outcome reporting across ADPKD trials, leading to improvements in the reliability and relevance of trial-based evidence to inform decisions about treatment and ultimately improve the care and outcomes for people with ADPKD.

## Background

Autosomal dominant polycystic kidney disease (ADPKD) is the most common inherited kidney disorder and is responsible for 5–10% of end-stage kidney disease (ESKD) requiring renal replacement therapy [1–3]. ADPKD is life-threatening and irreversible, and affects an estimated 12.5 million people worldwide [4] occurring equally in men and women, without ethnic disparities [5, 6]. Gene defects which lead to the development of ADPKD cause disruption in renal tubular epithelial differentiation leading to the formation of multiple cysts, and their expansion due to ongoing cellular proliferation and fluid secretion [7, 8]. The cysts progressively grow, enlarging kidneys up to 20 times bigger than normal size [9], displacing and destroying normal kidney tissue culminating in fibrosis and ultimately kidney failure [7, 8]. The rate of decline in kidney function is highly variable amongst patients (i.e. annual rate of decline in creatinine clearance 1.71 to 5.8 mL/min/1.73 m^2^), with greater reported rate of reduction in those who progress to ESKD [10, 11]. Up to 70% of patients with ADPKD progress to ESKD by the age of 65 years [12], and the annual cost of renal replacement therapy (RRT) provision for ADPKD patients in Europe alone approximates to 1.5 billion Euros [13]. Patients with ADPKD are also at high risk of complications including debilitating kidney pain, hepatic cysts, infection, intracranial aneurysms, and cardiovascular disease that may severely impair quality of life and well-being.

The past decade has witnessed some therapeutic advances, with treatments targeting proliferation of cysts [14] and cyst growth [15]. Despite the growing number of trials in ADPKD, the outcomes that are relevant to patients and clinicians, such as symptoms and quality of life, are infrequently and inconsistently reported [16]. Typically, patients are not included in the selection of outcomes and this may cause a mismatch in priorities between patients and clinicians [17]. For example, patients with ADPKD identified the psychosocial impact of diagnosis and the progression to ESKD as important outcomes, but these were not identified by health professionals on the guideline working group [17]. Pain, financial impact, stress, and anxiety are important concerns amongst patients but are inconsistently reported in trials. Although selected larger sized trials have reported quality of life-related outcomes, such as pain [15, 18], most trials have focused their attention on reporting biochemical and imaging parameters such as kidney function (e.g. estimated glomerular filtration rate (eGFR), serum creatinine concentration, doubling of serum creatinine or novel biomarkers) [14, 19–22] and kidney structure (e.g. kidney and cyst volumes) [14, 21, 22]. There are new initiatives currently being developed to improve research quality (e.g. PKD Outcomes Consortium, https://c-path.org/programs/pkd/) and tools to better capture ADPKD-specific health-related outcomes (e.g. ADPKD-Impact Scale) [23].

Other problems with outcome reporting include variability in the outcomes measured and reported among trials, and the potential outcome reporting bias. The heterogeneity of outcomes can jeopardise the ability to compare and combine trial results and reliably estimate relative effectiveness [24]. In a systematic review on the prevention of progression of chronic kidney disease (CKD) in patients with ADPKD, changes in kidney function were reported using serum creatinine (12 trials), GFR (13 trials), doubling of creatinine (4 trials), or need for RRT (2 trials) [16]. Only one or two studies were able to be included in each meta-analysis per outcome because of variability in the definitions adopted [16]. Without a core outcomes set routinely reported in all trials, selective reporting of outcomes found to favour the intervention may occur, leading to an overestimation of the true effect of the intervention, or failure to report adverse events which can expose patients to unrecognised risks of harm [25–27].

The increasing recognition of inefficiency and waste in medical research, attributed to problems with outcome selection [28], has given rise to numerous initiatives across medical specialties worldwide to develop core outcome sets—defined as an agreed minimum set of standardised outcomes that should be measured and reported in all trials on a specific clinical topic [29, 30]. This does not preclude trialists adding other outcomes specific to the trial population and intervention. The first of these initiatives was the Outcome Measures in Rheumatology (OMERACT), which was formed in 1992 to identify core outcomes in rheumatology through a consensus process involving healthcare providers, policy makers, patients, and their caregivers [31–33]. The implementation of OMERACT core outcomes has improved the reporting and relevance of outcomes in rheumatology trials [33–35]. More recently, the Core Outcome Measures in Effectiveness Trials (COMET) initiative was formed in 2010 to facilitate the development and implementation of core outcome sets [36, 37].

In nephrology, the Standardized Outcomes in Nephrology (SONG; www.songinitiative.org) initiative was formed in 2015 to establish core outcomes across the full spectrum of CKD. Ongoing work is focussed on haemodialysis (SONG-HD) [38–40], kidney transplantation (SONG-Tx) [41], peritoneal dialysis (SONG-PD), and paediatric CKD (SONG-Kids) [42]. The SONG-Polycystic Kidney Disease (SONG-PKD) project aims to establish a core outcome set for trials and other types of clinical research involving patients with ADPKD. The initial focus of SONG-PKD will be on ADPKD as it is the most common form of PKD and presents differently to autosomal recessive polycystic kidney disease (ARPKD). The SONG-PKD Steering Group was convened in June 2017 and is comprised of a multidisciplinary team of health professionals and patients with PKD. The aims of SONG-PKD are to: describe the scope and consistency of outcomes reported in trials in ADPKD; identify and prioritise outcomes that are important to patients with ADPKD and their caregivers (including the reasons for their choices); generate insights on health professionals’ perspectives on outcomes in ADPKD; develop a consensus-based set of core outcomes of critical importance to all stakeholder groups including patients/caregivers and health professionals; and establish a set of core outcome domains to be reported in all trials in patients with ADPKD.

## Methods/design

**Fig. 1:**
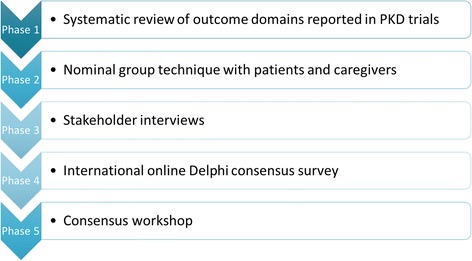
SONG-PKD study flowchart. *PKD* polycystic kidney disease

The SONG-PKD project will follow the SONG methodology [43], which has been adapted from the OMERACT and COMET initiatives [31, 36]. The five phases will include: a systematic review, focus groups with nominal group technique, semi-structured interviews with stakeholders, an international Delphi survey, and a consensus workshop (Fig. 1).

### Phase 1: Systematic review of outcome domains reported in PKD trials

We will conduct a systematic review to identify and assess the scope and consistency of outcome domains and outcome measures reported in randomised controlled trials (RCTs) of interventions for adults with ADPKD.

#### Study search strategy

A comprehensive search of MEDLINE, Embase, the Cochrane Kidney and Transplant Specialized Register, Australian and New Zealand Clinical Trials Registry (ANZCTR; www.anzctr.org.au), EU clinical trials register (www.clinicaltrialsregister.eu) and ClinicalTrials.gov will be conducted to identify all RCTs that enrolled patients with ADPKD aged 18 years or older. We will not apply date or language restrictions.

All RCTs published in peer-reviewed journals and trial protocols registered in ClinicalTrials.gov, ANZCTR, and EU clinical trials register will be included (up to 2 March 2017). Abstracts and conference reports will not be included as they do not provide a complete and reliable source of all the outcomes reported and measured in trials.

#### Types of interventions

Any intervention used to treat and manage adult patients with ADPKD will be included. These may include pharmacological, surgical, lifestyle, psychosocial, and health service interventions.

#### Types of participants

Adult patients with ADPKD aged 18 years or older will be eligible. Studies that exclude patients with ADPKD, or only enrol children (aged 18 years or below) with ADPKD will not be included. Given the focus on ADPKD, trials in which patients with ADPKD comprise less than half the study population across all arms will be excluded.

#### Eligibility of studies

All records retrieved from the electronic searches will be independently assessed by two reviewers (BS and YC). The full texts of all potentially relevant RCTs will be independently assessed by the two reviewers (BS and YC). Any disagreement on the eligibility of studies will be resolved through discussion with a third reviewer (AT).

#### Data extraction

Characteristics from all included trials will be extracted by one reviewer (BS) and will include the following: first author, publication date, country in which the trial was conducted, participant characteristics (age, sex), trial duration, name and type of intervention (e.g. pharmacological, psychosocial, lifestyle), and primary or secondary outcomes as reported in the trial (including definitions, measurement instruments, thresholds, measurement time points or time frames, changes in level or percentage, scores) [37–44]. Outcomes reported in the registration for each trial published will be evaluated in order to assess the selective reporting of outcomes. Two reviewers will cross check the data extraction (BS and YC).

#### Data analysis and presentation

The reviewer (BS) will group similar outcomes into outcome domains which will be classified as surrogate, clinical, or patient-reported. A surrogate endpoint or outcome is a biochemical or imaging marker used to substitute for a clinical outcome (e.g. total kidney volume) [45–47]. A clinical outcome is a medical outcome that is determined or diagnosed by the clinician (e.g. infection) [40, 48, 49]. Patient-reported outcomes are reported directly from patients regarding how they function or feel in relation to a health condition and its therapy, without interpretation by a healthcare professional or anyone else (e.g. pain). The domains will be reviewed and discussed by the SONG-PKD Steering Group. The frequency of reporting across trials for each outcome domain will be ascertained. The primary outcome, if specified, will be identified and analysed. The number of different outcomes (including outcome measures and measurement time points) and the number of trials that assessed each specific outcome will also be assessed. Statistical analyses will be performed using R version 3.2.3 (R Foundation for Statistical Computing, Vienna, Austria).

### Phase 2: Nominal group techniques with patients and caregivers

Patients with ADPKD and their caregivers (including family members) will identify and rank outcomes that they consider are important to include in trials and will discuss reasons for their choices. The nominal group technique is highly recommended as a transparent, equitable, and systematic approach [50–52] to generate ideas and consensus on priorities in health, including outcomes in CKD [53, 54], and allows each participant to raise their views and suggestions without direct rejection or criticism from others in the group [52].

#### Participants and recruitment

Patients with ADPKD aged 18 years and older and their caregivers will be eligible to participate. We will aim to convene a minimum of 20 nominal groups (involving 8–12 participants per session, estimated total *n* = 200). The final number of groups will depend on when data saturation, defined as the point when few or no new outcomes or issues are emerging, is reached [54–56].

Participants will be recruited initially from participating centres across Australia (Westmead Hospital, Princess Alexandra Hospital, Monash Medical Centre), and will aim to recruit sites in other countries upon funding securement.

We will use a purposive sampling strategy to achieve maximum diversity in demographics (age, sex, socioeconomic status, ethnicity, location, and educational attainment) and clinical characteristics (stages of chronic kidney disease; i.e. patients not on RRT, CKD stage 1–5; patients on haemodialysis or peritoneal dialysis, CKD stage 5D; and patients with a kidney transplant, CKD stage 5 T; time since diagnosis, comorbidities, and complications). Informed consent will be obtained from all participants. Each participant will be reimbursed in cash (A$50) to help support their transport to attend the session.

#### Data collection

Each focus/nominal group will be 2 h in duration and take place in a centrally located venue external to the hospital in order to encourage open discussion and to minimise censoring of conversation among participants due to feeling disempowered in a clinical setting. The question guide will be developed based on those previously used to elicit patient-prioritised outcomes in kidney transplantation and haemodialysis [53, 54], and with input from the SONG-PKD Steering Group and investigators. Each group will cover the following:

*Welcome and introduction (10 min):* The facilitator will explain the aims of the study, define what outcomes are in the context of clinical trials, and ask participants to introduce themselves.

*Focus group discussion (40 min):* Participants will be asked to discuss their experiences of living with ADPKD, including perceived benefits, harms, and complications related to the disease and treatment.

*Nominal group technique (70 min):* Each participant will be asked to suggest one or two outcomes they consider are the most important to be reported in trials in ADPKD. The facilitator (YC, BS, AT, TG) will write all suggested outcomes on the flipchart/board and ask the group to provide clarification as required or to discuss their reasons for their suggestion. Once the group has generated the outcomes, the facilitator will add to the flipchart/board outcomes identified from the systematic review (Phase 1) and in previous nominal groups. The list of outcomes will be discussed to ensure that all members understand the meaning of each outcome. A copy of the outcomes will be printed for participants to individually rank all the outcomes in the order of perceived importance, from 1 (most important) to X (least important). The facilitator will ask participants to read out their top three and note these on the flipchart/board. Similarities and differences in ranking will be discussed among the group.

All discussions during the session will be audiotaped and transcribed verbatim, and a note-taker will record the contextual details around the discussion.

#### Data analysis

##### Quantitative analysis

A measure of importance (i.e. importance score) of each outcome, based on the rankings attributed in the focus/nominal groups, will be used to prioritise the outcomes. The calculation of this measure is described as follows.

The distribution of the ranking for each outcome is obtained by calculating the probability of each rank for each outcome. Using mathematical notation, this is written as *P*(*O*_*j*_ *in rank i*), i.e. the probability of the outcome O_j_ being assigned the rank i. Thus, for each outcome we obtain the probability of being ranked in first place, in second place, and so on. By the total law of probabilities, these probabilities can be decomposed as:

$$ {\displaystyle \begin{array}{l}P\left({O}_j\kern0.5em in\kern0.5em \mathit{\operatorname{rank}}\;i\right)\kern0.5em =\\ {}\kern9.5em =\kern0.5em P\left({O}_j\kern0.5em in\kern0.5em \mathit{\operatorname{rank}}\;i\kern0.5em \left|{O}_j\kern0.5em is\kern0.5em nominated\right.\right)\kern0.5em \times \kern0.5em P\left({O}_j\kern0.5em is\kern0.5em nominated\right)\\ {}\kern9.5em +\kern0.5em P\left({O}_j\kern0.5em in\kern0.5em \mathit{\operatorname{rank}}\;i\kern0.5em \left|{O}_j\kern0.5em not\kern0.5em nominated\right.\right)\kern0.5em \times \kern0.5em P\left({O}_j\kern0.5em not\kern0.5em nominated\right)\end{array}} $$

where “nominated” means that the outcome was considered (and given a rank) by the participant. We will assume that the *P*(*O*_*j*_ *in rank i* |*O*_*j*_ *not nominated*) is 0. The reasoning for this is that if the participant did not mention the outcome *O*_*j*_, then the probability of any rank is 0. Therefore, the expression above simplifies to

$$ P\left({O}_j\kern0.5em in\kern0.5em \mathit{\operatorname{rank}}\;i\right)\kern0.5em =\kern0.5em P\left({O}_j\kern0.5em in\kern0.5em \mathit{\operatorname{rank}}\;i\kern0.5em \left|{O}_j\kern0.5em is\kern0.5em nominated\right.\right)\kern0.5em \times \kern0.5em P\left({O}_j\kern0.5em is\kern0.5em nominated\right) $$

From this expression, we can observe that the probability has two components: 1) the importance given to the outcome by the ranking; and 2) the consistency of being nominated by the participants. We then use these probabilities and compute the weighted sum of the inverted ranking $\left(\frac{1}{i}\right)$ to obtain the importance score (IS)

$$ IS\kern0.5em =\kern0.5em \sum \limits_{i=1}^{\begin{array}{l}\kern1.5em nr\kern0.5em of\\ {} outcomes\end{array}}P\left({O}_j\kern0.5em in\kern0.5em \mathit{\operatorname{rank}}\kern0.5em i\right)\kern0.5em \times \kern0.5em \frac{1}{i} $$

The importance score can be interpreted as a summary measure of importance of the outcome that incorporates the consistency of being nominated and the rankings given by the participants. The reason for inverting the ranks is to give more weight to top ranks and less to lower ranks. Higher values of the score identify outcomes that are more valued by the participants. The standard errors for the importance score can be obtained through bootstrapping. This measure has a similar motivation to the Expected Reciprocal Rank Evaluation Metric that was proposed in a different context [57].

The importance scores will also be calculated separately for patients and their caregivers/family*,* and these results will be compared using a t test with a statistical significance level of p < 0.05. The analysis will be conducted using the software package Stata/SE version 14.0 (StataCorp.*,* College Station, TX) and the R version 3.2.3 (R Foundation for Statistical Computing, Vienna, Austria).

##### Qualitative analysis

The transcripts will be imported into HyperRESEARCH (ResearchWare Inc., www.researchware.com, version 3.7.2) software to facilitate qualitative data analysis. The transcripts will be reviewed line by line to identify concepts, and similar concepts will be grouped into themes that reflect the reasons for identifying and ranking the outcomes. The preliminary findings will be discussed among the research team to ensure that themes reflect the full breadth and depth of the data.

### Phase 3: Stakeholder interviews

Semi-structured interviews will be conducted with health professionals to capture the range and depth of individual values, beliefs, and attitudes towards outcomes in PKD [56, 58]. We will use the Consolidated Criteria for Reporting Qualitative Health Research (COREQ) [59] to report this study.

#### Participants and recruitment

Health professionals (nephrologists, hepatologists, surgeons (i.e. urologists, transplant surgeons), geneticists, nurses, and allied health professionals (i.e. psychologists, social workers, genetic counsellors, dietitians)) who have expertise, experience, and interest in ADPKD will be eligible to participate in an interview. A minimum of 50 participants will be recruited worldwide through the networks of the Steering Group and investigators. We will apply a purposive sampling strategy to ensure a broad spectrum of perspectives by maximising variability in demographics, professional role, and experience. We will also specifically identify key informants who have experience in research (trials) and roles in policy making. Recruitment will continue until data saturation has been achieved. All participants will provide informed consent prior to the interview [55, 56, 60].

#### Data collection

Results from the systematic review (Phase 1) and nominal group techniques (Phase 2) will inform the design of the interview guide. We will conduct face-to-face interviews unless video-conferencing or telephone interviews are preferred by the participants, or when an in-person interview cannot be feasibly organised. Participants will be asked to reflect and discuss their perspectives on: 1) their role and experiences in providing care for patients with ADPKD, 2) aspects of treatment or care that are challenging, 3) shared-decision making in the context of ADPKD, 4) outcomes they consider to be critical or relevant to include in PKD trials and their reasons, 5) the results obtained from the nominal group technique study (Phase 2), and 6) views on the development and implementation of core outcomes in PKD trials. Each interview will take approximately 40 min, and will be audio-recorded and transcribed.

#### Data analysis

We will extract a list of outcomes suggested by the participants. Thematic analysis as described in Phase 2 of the project will be used to identify themes that reflect the attitudes, beliefs, and perspectives of the participants. Four investigators (YC, BS, TG, and AT) will be involved in the data analysis and review preliminary findings to ensure that the coding framework captures the full range and depth of the data collected. The preliminary findings will also be sent to all participants (i.e. member checking) for feedback and comment, and to add relevant perspectives as needed. This can enhance the analytical framework and ensure that the results reflect the perspectives of the participants.

### Phase 4: International online Delphi consensus survey

We will conduct an international online Delphi survey to gain consensus on the outcome domains that are critically important to all stakeholder groups for trials in ADPKD. The Delphi survey has been successfully used to reach consensus on core outcome sets for various health conditions or treatments [61–64], including in haemodialysis and kidney transplantation [65, 66]. The Delphi technique usually involves three rounds of surveys completed sequentially and anonymously by a panel of experts with experience or expertise on the topic of interest [30, 51]. Respondents contribute their individual perspectives (for example, rating the importance of an outcome) and, in subsequent rounds, participants can view their previous scores and the results of the group, reflect on the group results, and have the option of revising their opinion. This process results in equitable contribution from all participants as it allows individual respondents to express independent thought and minimises direct confrontation. Previous SONG Delphi surveys have demonstrated that participants do not appear to be unduly influenced by the group results (for example, increases in scores occur in both the patient/caregiver and health professional groups), and the scores of both stakeholder groups converge over the three rounds.

#### Participants and recruitment

The majority of Delphi studies used to develop core outcomes across medical specialties have reported sample sizes ranging from 13 to 222 participants. Over 1000 participants from more than 70 countries have participated on the Delphi panel for the studies conducted as part of the SONG-HD and SONG-Tx project [65, 66]. For the SONG-PKD Delphi panel, we will aim to recruit a minimum sample size of 1000 respondents where patients/caregivers comprise at least 50% of the sample size. Specifically, we will aim to recruit patients/caregivers (*n* = 500), clinicians (nephrologists/surgeons (i.e. urologists, transplant surgeons)/geneticists (*n* = 400)), nurses and allied health professionals (psychologists, genetic counsellors, and dietitians (*n* = 60)), researchers (n = 20), industry (*n* = 20), and policy makers (*n* = 20).

We will use multiple sampling strategies to be as broadly inclusive as possible. Participants will be identified by using a purposive strategy similar to those detailed in Phase 3 to ensure broad representation of views and snowballing whereby participants can nominate other eligible individuals to participate. Participants will be recruited globally through participating university institutions/hospitals of the SONG-PKD Steering Group and investigators, the SONG Initiative database, and patient/consumer organisations. All participants will be required to register their name and email address via www.songinititative.org to receive a standard study information sheet. Informed consent will be obtained from all participants.

#### Data collection

The Delphi survey will include approximately 30 outcome domains identified from Phases 1–3 of SONG-PKD. Each outcome will include a definition written in plain language. The draft Delphi survey will be reviewed by the SONG-PKD Steering Group and pilot tested with at least 10 patients/caregivers. The survey will be custom programmed and administered online using Qualtrics. All participants will be assigned a unique identifier based on their name and email address to link their responses across all three rounds of the Delphi survey. At least two reminders will be sent to participants per round. We aim to retain at least a 70% response rate across all rounds.

##### Round 1

Participants will rate the importance of each outcome domain using the GRADE nine-point Likert scale [67]. Ratings of 1–3 indicate outcomes of limited importance, ratings of 4–6 indicate outcomes of importance but not critical, and ratings of 7–9 indicate outcomes of critical importance. An option “unable to score” will also be available. In order to minimise the risk of ordering bias, the outcomes domains will be randomised. For each outcome, a text box is provided for participants to provide comments about their choices. After the rating scales are completed, participants can suggest new outcomes. All outcome domains that are suggested by more than 10% of the participants, and do not duplicate outcomes in the original survey, will be included in Round 2.

**Fig. 2:**
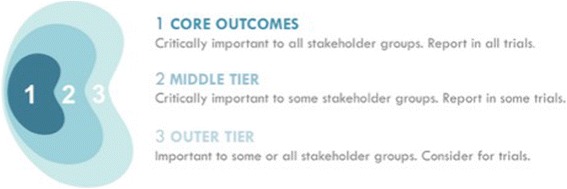
SONG conceptual schema of core outcomes

We will review the distribution of scores across all outcomes for each stakeholder group*,* i.e. patients/caregivers and health professionals. Any outcomes with a median and mean of more than 7 will be retained for Round 2. Any new outcomes suggested by more than 10% of the participants will also be included in the next round. This is based on criteria established for previous SONG Delphi surveys [38, 66]. Any outcomes excluded in subsequent rounds will be listed as “outer tier” outcomes*,* i.e. important to some or all stakeholder groups to consider for trials; or “middle tier” outcomes*,* i.e. critically important to some stakeholder groups to report in some trials (Fig. 2).

##### Round 2

The participants will be shown their previous score for each outcome (highlighted in the rating scale), and will be presented with a column graph of the distribution of scores for all participants (equally weighted by stakeholder group), patients/caregivers, and health professionals. A plain language explanation and an example will be provided to help participants understand how to interpret the graph. Participants will also see comments from patients/caregivers and health professionals in separate scroll down boxes. Participants will be asked to re-rate the outcomes on the same GRADE 9-point Likert scale. A text box will be provided for optional comments such as reasons for their ratings.

An outcome with a median and mean of more than 7, and with 70% or more participants in both stakeholder groups (i.e. patient/family member and health professionals) rating the outcome to be of critical importance (7–9), will be included in Round 3.

##### Round 3

In this final round of survey, participants will see their previous score highlighted and again review the distribution of scores for all participants, patients/caregivers, and health professionals, and comments from Round 2. They will be asked to re-rate the importance of each outcome, and have the opportunity to provide additional comments in a free-text box. To assess the relative importance of the outcomes, we will include a Best–Worst Scale survey [68]. Participants will be presented with approximately six blocks of six outcome domains in which they select the most important and the least important outcome from the list.

#### Data analysis

For all three rounds, we will show the distribution of scores and calculate the mean, median, and proportion of participants rating the outcome of critical importance. For the Best–Worst Scale Survey, multinomial logistic regression models will be used to calculate the relative importance score for each outcome domain normalised to the range of 1 (least important) to 9 (most important). This will be calculated separately for patients/caregivers and health professionals.

For feasibility, three to five outcome domains are included in the core set and all other outcomes are classified as middle and outer tier outcomes (Fig. 2). It is possible that more than five outcomes may be identified based on the SONG pre-specified definition of consensus. For outcomes to be included in the core set, the outcome must have: a median score of greater than or equal to 8; a mean score greater than or equal to 7.5; the proportion of participants rating the outcome as ‘critically important’ being greater than or equal to 75%, and a median score of less than 10 in the forced ranking question [65, 66]. As we are unable to determine the distribution of scores, the thresholds for inclusion of an outcome domain as a core outcome may need to be determined post-hoc and discussed at the consensus workshop (Phase 5). Several subgroup analyses will be conducted according to country, gender, CKD stage, and primary role (e.g. patient/caregiver, health professionals).

### Phase 5: Consensus workshop

A face-to-face stakeholder consensus workshop will be convened to obtain feedback and discuss the proposed core outcomes for trials in PKD as identified from Phases 1–4. The meeting will be chaired and facilitated by members of the SONG-PKD Steering Group, and held in conjunction with an international nephrology conference to maximise participation. Based on previous consensus workshops, and to ensure feasibility and group manageability [40, 69], we will invite approximately 60 participants, including at least 20 patients with ADPKD and their caregivers. Health professionals (nephrologists, surgeons, geneticists, nursing, allied health professionals, researchers, policy makers, and industry) with a range of clinical and research experience in ADPKD, including in clinical trials, or who have leadership or advisory roles in major research, funding, and regulatory agencies will be invited to attend. Patients and caregivers attending the workshop will be reimbursed for transportation and parking.

A copy of results from Phases 1–4 will be provided to participants approximately 2 weeks before the workshop so that participants can have the opportunity to ask questions or seek clarification by email, and understand the SONG-PKD process and results to inform the discussion. The workshop will comprise of three sessions.

#### Session 1: Introduction

We will present an introduction to the SONG-PKD initiative, including the process and results from the Phases 1–4, and the proposed set of core outcome domains with the threshold and rationale for inclusion.

#### Session 2: Breakout group discussion

Participants will be allocated to six breakout groups with up to 12 participants in each group (including a facilitator and co-facilitator). Each group will include at least three patients/family members/caregivers to encourage exchange of different perspectives and opinions, and breadth of discussion. All facilitators will attend a briefing session and be provided with a question guide prior to the session commencement. Participants will be asked to reflect on and discuss their opinions on the proposed core outcome domains.

#### Session 3: Plenary discussion

The Chair of the workshop will moderate a plenary discussion. A nominated spokesperson from each breakout group will present a summary of the discussion to the wider group who will have the opportunity to respond and discuss the issues raised by other groups. The Chair will summarise the key points raised across all breakout groups.

All breakout and plenary discussion will be audiotaped and transcribed verbatim. An investigator (YC) will summarise the discussion with input from other investigators (BS, AT, and TG) to ensure that the summary is comprehensive and captures all the points raised in the discussion. A draft plain language report will be circulated to the workshop attendees and collaborators for feedback within a 2-week timeframe. The readability of the plain language report will be at the sixth-grade level, which will be of lower literacy level than the eight-grade level as recommended by the Centers for Disease Control and Prevention (CDC) [70]. Additional comments will be integrated into the final report.

### Establishing the SONG-PKD core outcome domains

The SONG-PKD proposed core outcomes will be uploaded on the website for 3 weeks for public comment. The link will also be sent to the SONG Initiative database and collaborating organisations. All feedback received will be reviewed by the SONG-PKD Steering Group to finalise the SONG-PKD set of core outcome domains.

### Ethics

This study has been approved by the Human Research Ethics Committee of The University of Sydney (2015-228), Westmead Hospital (2009/6/4.14), Monash Medical Centre (2010.0.31), and Princess Alexandra Hospital (HREC/17/QPAH/112).

## Discussion

The SONG-PKD project engages patients/caregivers and health professionals in a systematic, transparent, and equitable consensus process to establish a set of critically important core outcome domains to be reported in all trials involving patients with ADPKD. We will establish validated, feasible, and robust core outcome measures for each core outcome domain identified through SONG-PKD.

Reporting outcomes that are critically important to all stakeholder groups, particularly patients, will improve the quality and relevance of research evidence to inform treatment decisions. We will actively disseminate the core outcome domains through publications, online media, and collaborating patient, research, professional, and policy organisations, and develop targeted strategies to ensure implementation of the core outcome domains in trials. Standardised reporting of patient-important outcomes in clinical trials is expected to translate to improved patient care and outcomes in ADPKD.

## Study status

Recruitment and data collection have commenced.

## Abbreviations

ADPKD: Autosomal dominant polycystic kidney disease
ANZCTR: Australian and New Zealand Clinical Trials Registry
CKD: Chronic kidney disease
COMET: Core Outcome Measures in Effectiveness Trials
COREQ: Consolidated Criteria for Reporting Qualitative Health Research
eGFR: Estimated glomerular filtration rate
ESKD: End-stage kidney disease
OMERACT: Outcome Measures in Rheumatology
PKD: Polycystic kidney disease
RCT: Randomised controlled trial
RRT: Renal replacement therapy
SONG: Standardised Outcomes in Nephrology
SONG-PKD: Standardised Outcomes in Nephrology—Polycystic Kidney Disease
